# Greener pathway toward the synthesis of lichen‐based ZnO@TiO_2_@SiO_2_ and Fe_3_O_4_@SiO_2_ nanocomposites and investigation of their biological activities

**DOI:** 10.1002/fsn3.1661

**Published:** 2020-06-27

**Authors:** Shorish M. Abdullah, Kamal Kolo, S. Mohammad Sajadi

**Affiliations:** ^1^ Department of Biogeoscience Scientific Research Center Soran University Soran Iraq; ^2^ Department of Biology Faculty of Science Soran University Soran Iraq; ^3^ Department of Nutrition College of Health Technology Cihan University‐Erbil Kurdistan Region Iraq; ^4^ Department of Phytochemistry Scientific Research Center Soran University Soran Iraq

**Keywords:** biological activities, Fe_3_O4@SiO_2_, green synthesis, *Lecanora muralis*, ZnO@TiO_2_@SiO_2_

## Abstract

A green way is introduced to biosynthesis of ZnO@TiO_2_@SiO_2_ and Fe_3_O_4_@SiO_2_ nanocomposites using the bioactive potential of *Lecanora muralis* (LM) lichen. UV‐Vis spectroscopy and GC–Mass analysis of the lichen show the presence of various bioactive constituents inside the lichen aqueous extract. The XRD, SEM, EDS, and elemental mapping techniques revealed the well fabrication of biosynthesized nanostructures. Also, investigation of antibacterial and antifungal activities of nanostructures demonstrated that green synthesized nanostructures have a very good antibacterial ability against *Staphylococcus aureus*, *Escherichia coli*, *Pseudomonas* spp. and *Candida* spp. pathogenic bacteria, and fungi but no antifungal activity toward the *Aspergillus flavus*, *Aspergillus niger*, and *Aspergillus terrus* fungi species.

## Graphical abstract

This study investigates the greener pathway toward the synthesis of lichen‐based ZnO@TiO2@SiO2 and Fe3O4@SiO2 nanocomposites and investigation of their biological activities.

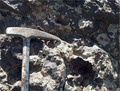

## 1 INTRODUCTION

Nowadays, synthetic nanomaterials are a great challenge for human life as they mostly produce using noneco‐friendly and expensive processes through harsh conditions. In some cases, applications of them are limited due to the adsorption of chemicals and hazardous materials on their surfaces such as their employments in medicinal usages; thus, currently researchers trying to replace and improve the traditional methods of nanomaterial synthesis to the green, economic, and safe methods, (Sajadi, Kolo, Abdullah, et al., 2018; Sajadi, Kolo, Hamad, et al., 2018; Scheringer, 2008; Shi, Magaye, Castranova, & Zhao, 2013; Zhang, Guo, Li, Wang, & Liu, 2018). Green routes of science and technology cover a broad area mainly involves the monitoring and assessment, pollution prevention and control, and remediation and restoration. In fact, tracking those pollutions is essential to avoid the production of environmentally hazardous substances or alter human activities in ways that minimize damage to the environment. In nanotechnology, both controlling the hazardous of nanomaterials before entering the environment and also improving the condition of ecosystem caused to introduce the concept of eco‐nanotechnology as an important line of the environmentally friendly technologies, (Nasrollahzadeh, Sajadi, & Hatamifar, 2016; Oomen et al., 2015; Scott‐Fordsmand et al., 2017; Sun et al., 2015; Vijayaraghavan & Ashokkumar, 2017).

Ever since the birth of mankind, human beings have been dependent on plants to fulfill their basic needs of life and even for the maintenance and restoration of health which among the plants, lichens are a wide range of habitats throughout the world, (Ismed et al., 2018; Ranković., Kosanić, & Stanojković, 2011; Walker., Lintott, 1997).

In fact, they are symbiotic organisms with no roots, leaves, or flowers and in a close symbiotic association consisting two unrelated organisms of fungal and photosynthetic partner such as green algae or cyanobacteria. Up to now, a lot of species of lichens and their products are known and directly or indirectly employed in medicinal and industrial usages. Among these compounds, secondary metabolites produced by lichens caused to their widely applications in folk and modern medicine due to their diverse bioactivities against various diseases such as usnic acid with a potential effect in cancer therapy due to its antimitotic and antiproliferative action. Lichen‐forming fungi produce antibiotic secondary metabolites that protect many animals from pathogenic microorganisms; thus, in this way they demonstrated a very good antibacterial activity. Screening the lichens has revealed the frequent occurrence of metabolites with antioxidant properties. In fact, they act as bioreducer to prevent the side effects of free radicals inside the human bodies. Beside the medicinal effects of antioxidant phytochemicals inside the lichens, they have a very good potential to reduce and stabilize metallic salts and convert them to the metal nanoparticles through an electron transfer mechanism, (Kumar, Kumar, & Kumar, 2016; Agbanelo, Adesalu, 2016; Nugraha et al. 2019; Jayanthi, Priya, Monica Devi, & Benila Smily, 2012; Santiago, Sangvichien, Boonprago, & Dela Cruz, 2013; Rankovic, Kosanic, 2015; Boustie, & Grube, 2005).

Therefore, during this study we used *Lecanora muralis* (LM) collected from Iraqi Kurdistan as the source of bioactive antioxidant phytochemicals to green synthesis of ZnO@TiO_2_@SiO_2_ and Fe_3_O_4_@SiO_2_ nanocomposites and evaluation of their antibacterial and antifungi activities.

## 2 MATERIALS AND METHODS

### 2.1 Instrumentations

All chemicals used in current study were purchased from Merck and Aldrich companies. XRD analysis was carried out for both lichen thalli and nanostructures to determine the types and formation of organic biominerals and the mineralogy of prepared nanostructures. The instrument used was PANalytical X'Pert^3^ Powder using Cu Kα radiation equipped with a diffractometer system XPERT‐PRO. The diffraction pattern was recorded for 2θ from 5° to 70° and a 2θ step scan of 0.010° was used, counting for 0.5 s at every step. The voltage and current of the generator were set at 45 kV and 40 mA, respectively. The prepared nanomaterials coated with gold using coater machine; then, particle morphology was investigated using FEI (Quanta 450) scanning electron microscopy equipped with Quantax EDS–Xflash 6/10 microanalyzer for detecting chemical composition of the prepared nanomaterials. The GC–MS analysis of bioactive compounds in both species was done using Agilent Technologies GC systems with GC‐7890B/MS‐5977 model comprising an 7693A automatic liquid sampler, equipped with HP‐5MS column (30 m in length × 250 μm in diameter × 0.25 μm in thickness of film). Spectroscopic detection by GC–MS involved an electron ionization system which utilized high‐energy electrons (70 eV). Pure helium gas (99.995%) was used as the carrier gas with flow rate of 1 ml/min, and an injection volume of 1 μl was employed in splitless mode. The injector temperature was maintained at 250°C, the ion source temperature was 200°C, and the oven temperature was programmed from 50°C (isothermal for 2 min), with an increase of 3°C/min to 150°C, then 10°C/min to 300°C, ending with a 5 min isothermal at 300°C. Mass spectra were taken at 70 eV: a scan interval of 0.5 s and fragments from 45 to 550 Da. The solvent delay was 0–3 min. Interpretation on mass‐spectrum GC‐MS was conducted using the database of National Institute Standard and Technology (NIST). UV–visible spectral analysis was recorded on a double‐beam spectrophotometer (Super Aquarius) to monitor the SPR signals of nanoparticles.

### 2.2 Study area

**Figure 1:**
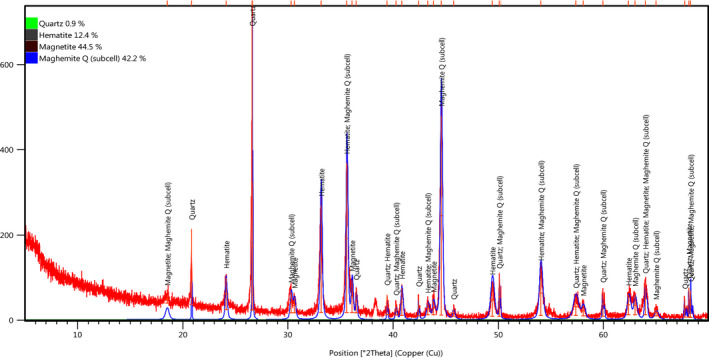
XRD analysis of the rock inhibiting studied lichens

The lichen was collected from Grdmandil mountain, Choman district, Erbil Provinces, located between 36°39.60 Latitude and 44°57.40 Longitude at the elevation 1,381 m above sea level. The main structure component of the of the rock‐inhabiting lichens in the studied area is Quartz, Hematite, Magnetite, and Maghemite Q (subcell) as shown in Figure 1.

### 2.3 Specimen (lichen) collection and identification

**Figure 2:**
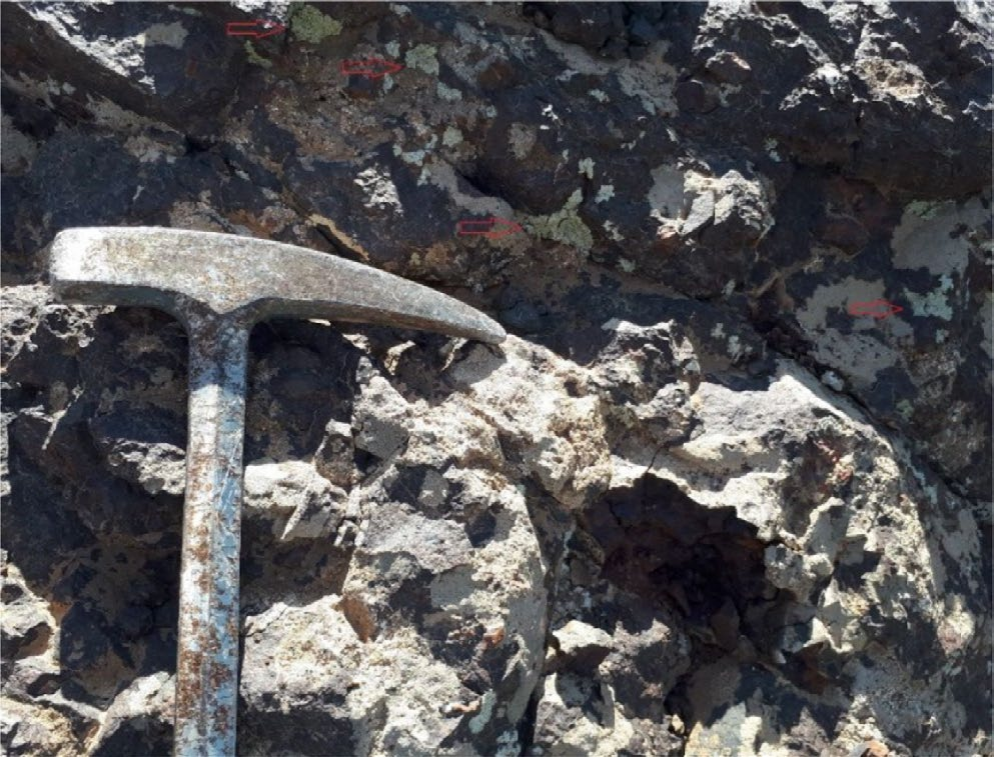
Field image of *Lecanora muralis (LM)* lichen

Specimen of lichen *Lecanora muralis* (*LM*) was collected with its rock sample. The species of lichen was identified previously in this area by Aziz (2005). Possible dust was carefully removed with soft brush on the lichen thalli then carefully detached from substratum with a sharp blade. Pretreated lichen was air‐dried then ground in a porcelain mortar to obtain the required amount for the analysis, Figure 2.

### 2.4 GC–Mass analysis of Lecanora muralis lichen species

**Figure 3:**
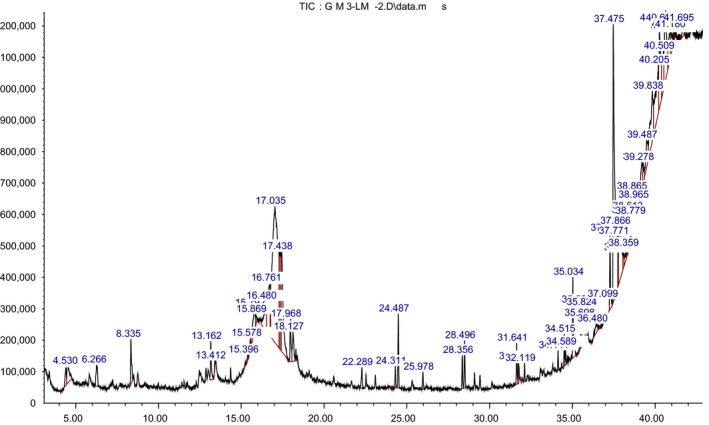
GC–Mass analysis of the extract of *Lecanora muralis lichen species*

The pulverized of lichen thalli *Lecanora muralis* submerged in methanol, incubated overnight with stirring on a magnetic stirrer, and filtered through Whatmann No. 41 filter paper; then, 1 μl of the sample of the solutions was employed in GC‐MS for analysis of different compounds, Figure 3. The GC–Mass analysis of the lichens showed the presence of various types of bioactive phytochemicals responsible for green synthesis of nanocomposites, Table 1.

**Table 1:** Phytochemicals monitored by GC‐Mass analysis of the extract of *Lecanora muralis lichen species*

RT (min)	Hit name	RT (min)	Hit name
4.532	Erythritol	35.034	Hexadecanoic acid, 2‐hydroxy‐1‐(hydroxymethyl)ethyl ester
25.976	Cyclononasiloxane, octadecamethyl‐	35.034	Hexadecanoic acid, 2‐hydroxy‐1‐(hydroxymethyl)ethyl ester
4.532	Erythritol	35.523	Bis(2‐ethylhexyl) phthalate
28.354	9,12‐Octadecadienoic acid, methyl ester	35.523	Bis(2‐ethylhexyl) phthalate
6.263	Undecane	35.698	2′‐Hydroxy‐5′‐methylacetophenone
28.494	9,12,15‐Octadecatrienoic acid, methyl ester,	35.698	2′‐Hydroxy‐5′‐methylacetophenone,
6.263	Undecane	35.826	2,5‐Dihydroxybenzoic acid
31.641	Undecanoyl chloride	35.826	2,5‐Dihydroxybenzoic acid,
8.333	Ethanol, 2‐phenoxy‐	36.479	Cyclotrisiloxane, hexamethyl‐
31.74	1,3‐Adamantanediacetamide	36.479	Cyclotrisiloxane, hexamethyl‐
8.333	Ethanol, 2‐phenoxy‐	37.097	2‐Ethylacridine
32.119	1,1,1,5,7,7,7‐Heptamethyl‐3,3‐bis(trimethylsiloxy)tetrasiloxane	37.097	2‐Ethylacridine
15.397	L‐Arabinitol	37.278	Octasiloxane, hexadecamethyl‐
34.142	Octasiloxane, hexadecamethyl‐	37.278	Octasiloxane, hexadecamethyl‐
15.397	L‐Arabinitol	37.476	Usnic acid
34.515	Tetrasiloxane, decamethyl‐	37.476	Usnic acid
15.578	Ribitol	37.773	(+)‐Usnic acid
34.591	Tetrasiloxane, decamethyl‐	37.773	(+)‐Usnic acid
15.578	Ribitol	37.866	1H‐Indole, 1‐methyl‐2‐phenyl‐
15.793	DL‐Arabinitol	38.094	Benzo[h]quinoline, 2,4‐dimethyl‐
15.793	DL‐Arabinitol	38.263	Cyclotrisiloxane, hexamethyl‐
15.869	D‐Mannitol	38.362	Arsenous acid, tris(trimethylsilyl) ester
15.869	D‐Mannitol	38.548	1,4‐Bis(trimethylsilyl)benzene
16.481	DL‐Arabinitol	38.612	4‐Methyl‐2‐trimethylsilyloxy‐acetophenone
16.481	DL‐Arabinitol	38.7	Phenylacetic acid, 2‐ethyl ester
16.726	L‐Arabinitol	38.781	Arsenous acid, tris(trimethylsilyl) ester
16.726	L‐Arabinitol	38.863	4‐(4‐Hyd.phenyl)‐4‐methyl‐2‐pentanone
16.761	Xylitol	38.962	2′‐Hydroxy‐5′‐methylacetophenone,
17.035	Sorbitol	39.201	4‐(4‐Hyd.phenyl)‐4‐methyl‐2‐pentanone,
17.297	Pentane‐1,2,3,4,5‐pentaol	39.277	Cyclotrisiloxane, hexamethyl‐
17.367	DL‐Arabinitol	39.487	4‐(4‐Hyd.phenyl)‐4‐methyl‐2‐pentanone,
17.437	Galactitol	39.836	Methyltris(trimethylsiloxy)silane
17.967	Benzoic acid, 2,4‐dihydroxy‐6‐methyl‐, methyl ester	40.204	Tris(tert‐butyldimethylsilyloxy)arsane
18.125	Butane, 1,2,3,4‐tetrachloro‐1,1,2,3,4,4‐hexafluoro‐	40.507	Methyltris(trimethylsiloxy)silane
22.286	Cyclononasiloxane, octadecamethyl‐	40.623	Methyltris(trimethylsiloxy)silane
24.309	7,9‐Di‐tert‐butyl‐1‐oxaspiro(4,5)deca‐6,9‐diene‐2,8‐dione	40.909	Silicic acid, diethyl bis(trimethylsilyl) ester
24.49	Hexadecanoic acid, methyl ester	41.696	Tetrasiloxane, decamethyl‐

### 2.5 Preparation of lichen extracts

The *Lecanora muralis* (LM) lichen was used to green synthesis of nanocomposites. 2 g *Lecanora muralis (LM)* was mixed to 30 ml distilled water at 80°C for 1 hr then filtered. The filtrate was used as lichen extract to biosynthesis of nanoparticles.

### 2.6 Green synthesis of Lecanora muralis (LM) based ZnO@TiO_2_@SiO_2_ and Fe_3_O_4_@SiO_2_ NCs

**Scheme 1:**
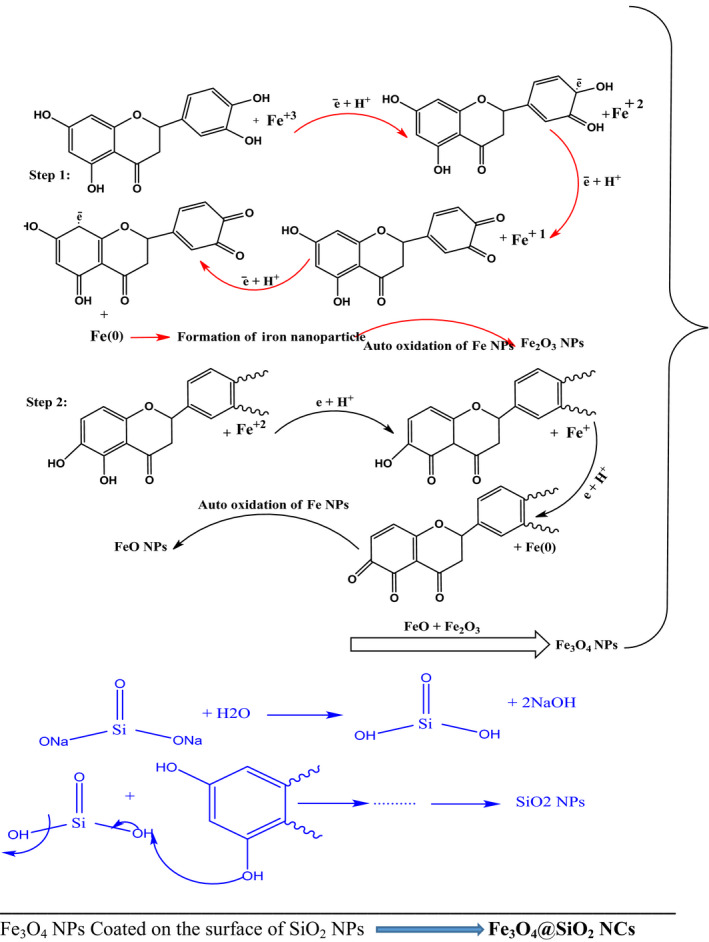
Biosynthesis mechanism of Fe_3_O_4_@SiO_2_ NCs using the antioxidant potential of *Lecanora muralis* extract

One pot green synthesis procedure was used during the current study. Initially, 0.5 g of ZnCl_2_, 1.5 g of titanyl hydroxide TiO(OH)_2_ and 2.5 g Na_2_SiO_3_ were mixed with 20 ml *LM* extract at pH 9 (as adjusted using Na_2_CO_3_) and 80°C under stirring for 5 hr until the formation of a slightly light precipitate of ZnO@TiO_2_@SiO_2_ NCs. The precipitate was separated using filtration, washed with hot distillate water to remove the impurities then dried, and kept to further investigations. In case of fabrication of Fe_3_O_4_@SiO_2_ nanocomposites, 0.7 g FeCl_2_ and 1.2 g FeCl_3_ were mixed with 20 ml *LM* extract containing 2 g Na_2_SiO_3_ at pH 9 (as adjusted using Na_2_CO_3_) while stirring at 80°C for 5 hr. In the next step, the precipitate was separated using filtration, washed with hot distillate water to remove the impurities then dried, and kept to further investigations, Scheme 1.

### 2.7 Bioactivity of lichen extract and prepared nanomaterials

For more accuracy, both disk diffusion and well diffusion methods were used to determine antibacterial activity. The antifungal (*Candida* spp.) activity of the lichen extract and nanostructures was monitored using the poisoned food method.

#### 2.7.1 Antibacterial activity

Disk diffusion method inoculated bacterial suspension 10^5^ CFU/ml of Mueller–Hinton agar. Sterile filter paper disks loaded separately with lichen extract and prepared nanomaterials were placed on the top of Mueller–Hinton agar plates. The plates were incubated at 37°C for 24 hr. The diameter of inhibition zone was measured after 24 hr incubation using a caliper and recorded in millimeters.

Well diffusion method: Three hundred microliters of microbe cultures of age 18–24 hr were added to Petri plates, and nutrient agar was poured. Once the medium was solidified, holes were made and each hole was packed with lichen extract and prepared nanomaterials separately. The plates were incubated at 37°C for 24 hr. The diameter of inhibition zone was measured after 24 hr incubation using a caliper and recorded in millimeters.

#### 2.7.2 Antifungal activity (Aspergillus spp.)

The poisoned food method was used in the preliminary screening of lichen aqueous extracts and prepared nanomaterials for their antifungal properties’ evaluation. First, the mycelia growths were evaluated in 60 mm Petri dishes filled with PDA solid medium amended with lichen extracts and prepared nanomaterials. Next, the center of each Petri dish was inoculated with 5 mm diameter disk of fungal mycelium, taken from pure culture (7 days old). Then, all inoculated dishes were incubated at 25°C for 6 days. After that, the radial mycelial growth was measured 6 days after inoculation. Finally, the antifungal activity of each extract was calculated in terms of inhibition percentage of mycelia growth by using the formula of:

%inhibition=(dc-dt)/dc×100

where *dc* is the average increase in mycelia growth in control, and *dt* is the average increase in mycelia growth in treated, (Hale, 1974; Singh, Tripathi, 1999).

## 3 RESULTS AND DISCUSSION

Lichens contain various secondary bioactive metabolites within the thalli and typically form crystals on the surface of the fungal hyphae. Their secondary metabolites have many pharmaceutical properties such as antimicrobial, antioxidant, antiviral, anticancer, antigenotoxic, anti‐inflammatory, analgesic, and antipyretic activities, Table 1. Hence, the present study is undertaken the potential ability of lichen extract as the source of antioxidants to biosynthesis of some bioactive nanostructures.

### 3.1 Identification of the green synthesized lichen‐based Fe_3_O_4_@SiO_2_ NCs

**Figure 4:**
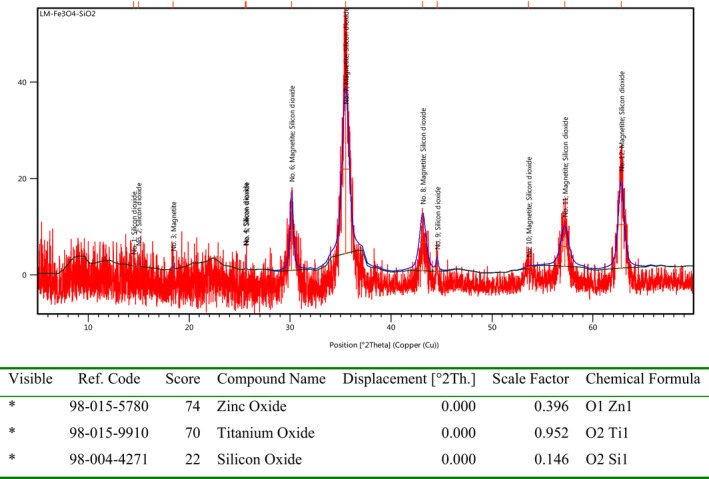
The XRD diffractogram of green synthesized lichen‐based Fe_3_O_4_@SiO_2_ NCs

According the XRD shown in Figure 4, it clearly reveals the presence of both magnetite and silicon dioxide nanoparticles. Of course, it should be considered that the SiO_2_ in its nature is an amorphous system but in the mentioned nanocomposite, the Fe_3_O_4_ NPs were coated on their surface; thus, we can see the silicon oxide nanoparticles as a crystalline system. Also, the XRD diffractogram confidently demonstrates the fabrication of Fe_3_O_4_@SiO_2_ NCs in a crystallinity pure and nanosized form. Moreover, Shearer's equation which revealed the size of nanoparticles around 53 nm.

**Figure 5:**
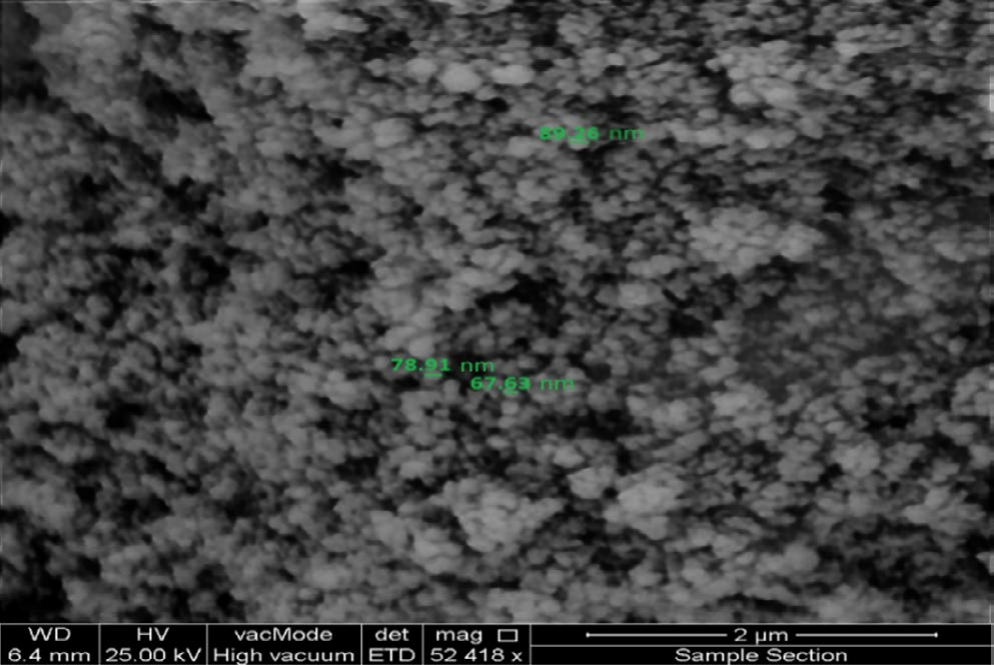
The SEM micrograph of green synthesized lichen‐based Fe_3_O_4_@SiO_2_ NCs

Figure 5 shows the SEM micrograph of Fe3O4@SiO2 NCs easily shows the size, shape, morphology, and homogeneity of nanoparticles. According the SEM image, the magnetite nanoparticles coated on the surface of silicon dioxide to form the Fe_3_O_4_@SiO_2_ NCs nanocomposite in a nanosized form ranging 55–90 nm and in a spherical shape and homogeneous morphology. This technique is also strongly confirming the fabrication of NCs. Of course, the SEM image shows some agglomerations which is a usual process for nanostructures due to their large surface area.

**Figure 6:**
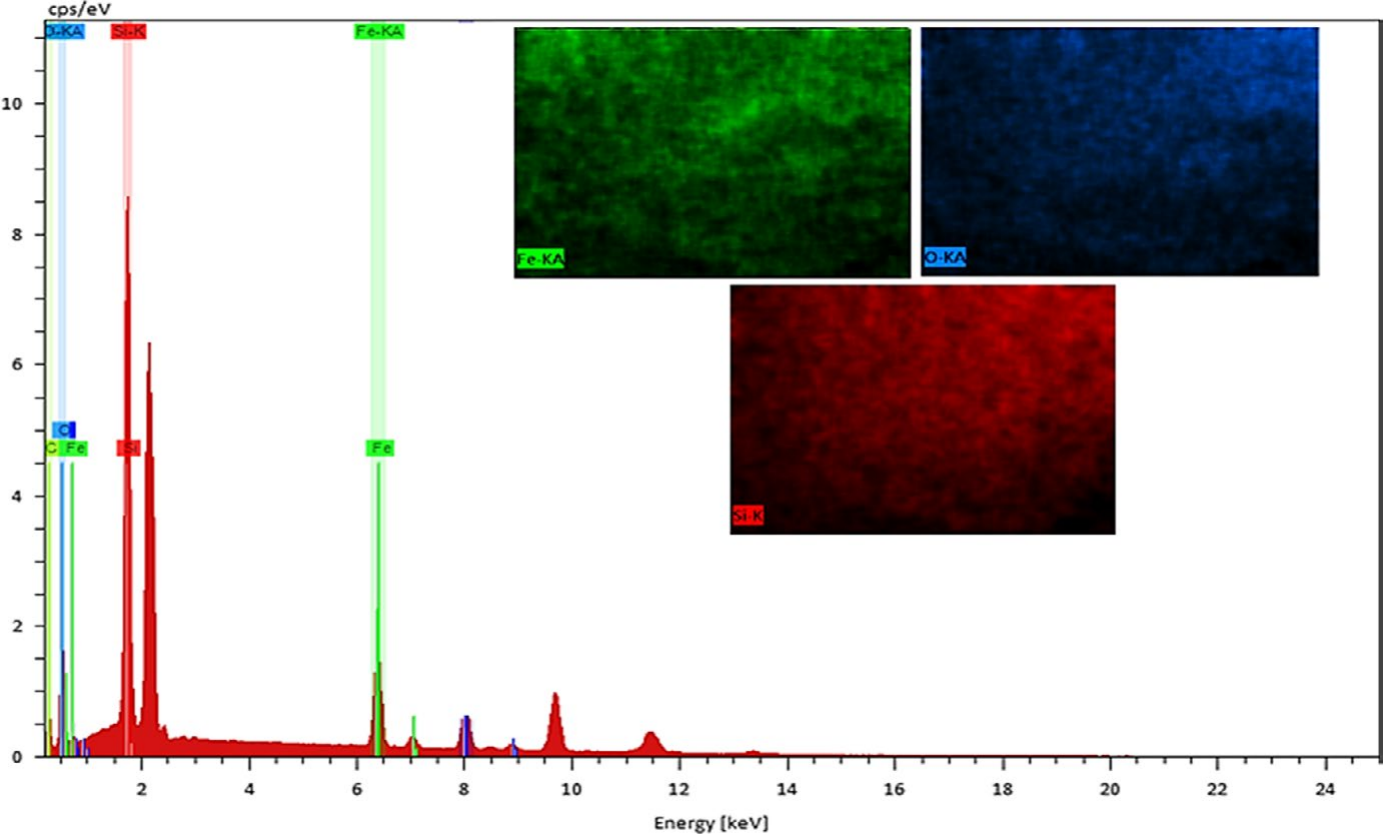
The EDS spectrum and elemental mapping of green synthesized lichen‐based Fe_3_O_4_@SiO_2_ NCs

Further, the EDS spectrum and elemental mapping represent the well‐defined peaks of compositional elements of NCs including Fe, O, and Si, thereby these results confirm the successful anchoring of the Magnetite on the surface of silicon dioxide NPs, Figure 6. Thus, all analysis showed the fabrication of the Fe3O4@SiO2 NCs.

### 3.2 Identification of the green synthesized lichen‐based ZnO@TiO_2_@SiO_2_ and Fe_3_O_4_@SiO_2_ NCs

**Figure 7:**
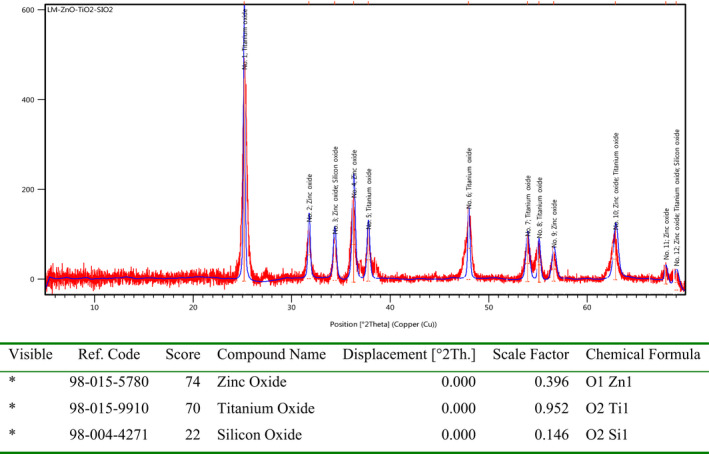
The XRD diffractogram of green synthesized lichen‐based ZnO@TiO2@SiO2 NCs

Besides the application of LM lichen to biosynthesis of Fe3O4@SiO2 NCs, it was also used to synthesis of bioactive ZnO@TiO_2_@SiO_2_ NCs as according the UV‐Vis and GC–Mass analysis this lichen is full of bioactive phytochemicals such as antioxidants. The XRD diffractogram of the ZnO@TiO_2_@SiO_2_ NCs in Figure 7 clearly shows the crystalline structure of the nanocomposite in a homogeneous and nanoregim. According the diffractogram, all signals are belonging to the nanoparticles within the structure of nanocomposite and there are no other signals indicating the impurities inside the NCs. Furthermore, the nanoscale size of the nanocomposite was elucidated using Shearer's equation which revealed the size of nanoparticles around 55 nm.

**Figure 8:**
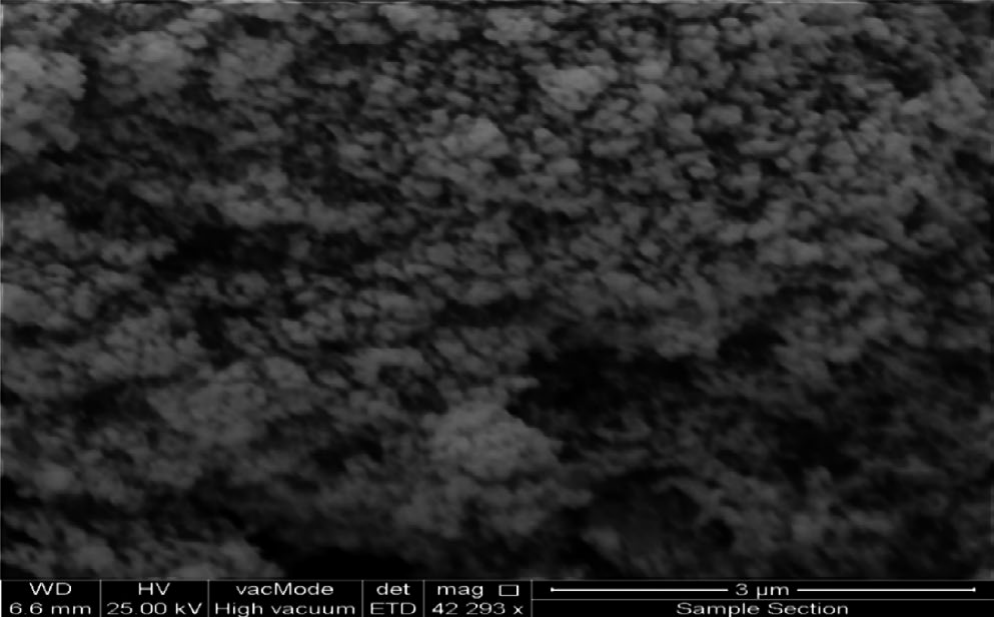
The SEM micrograph of green synthesized lichen‐based ZnO@TiO2@SiO2 NCs

**Figure 9:**
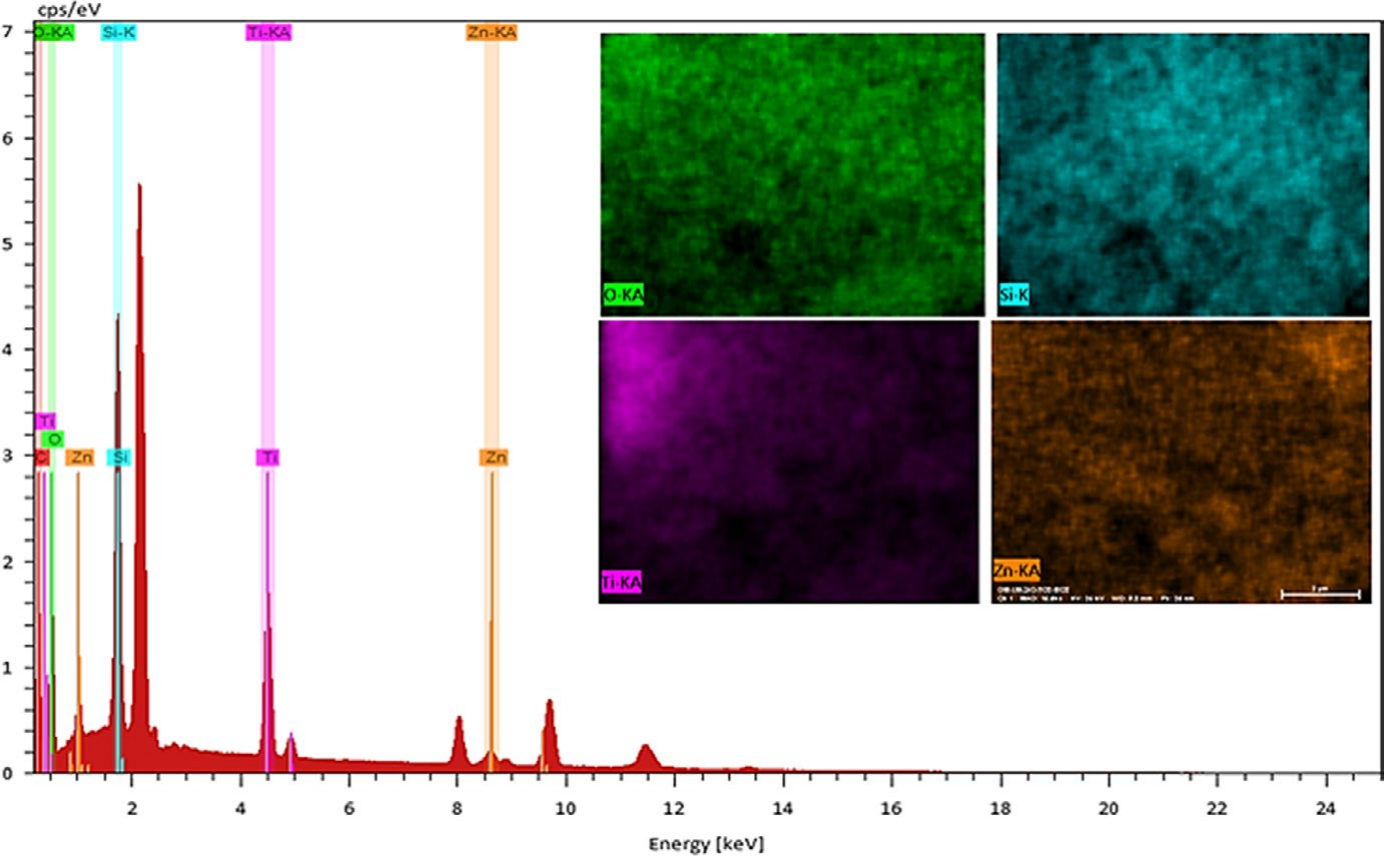
The EDS spectrum and elemental mapping of green synthesized lichen‐based ZnO@TiO2@SiO2 NCs

Besides the results obtained by XRD analysis for ZnO@TiO_2_@SiO_2_ NCs, the SEM, EDS, and elemental mapping of the NCs prove the structure of NCs as the SEM micrograph shows some agglomerated nanosized homogeneous and spherical shape nanostructure ranging 50–85 nm. Also, the EDS and mapping analysis of the nanocomposite demonstrated that it was fabricated from the Zn, O, Ti, and Si nanoparticles with no further elements indicating the presence of impurities inside the fabricated structure, Figures 8 and 9. Therefore, all analysis confirmed the fabrication of ZnO@TiO_2_@SiO_2_ NCs using the bioactive potential of the lichen.

### 3.3 Antimicrobial and antifungal activities of lichen extract and prepared nanomaterials

**Figure 10:**
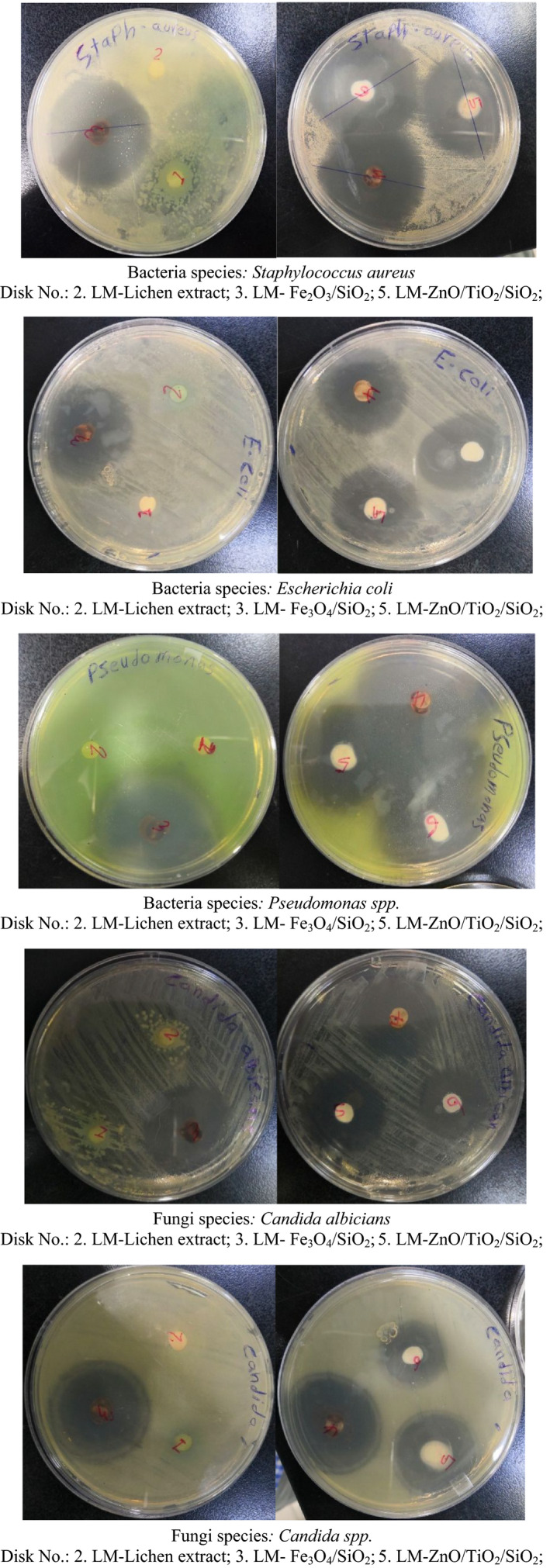
Antibacterial (disk diffusion method) and antifungal activities of LM lichen extract and green nanomaterials

**Figure 11:**
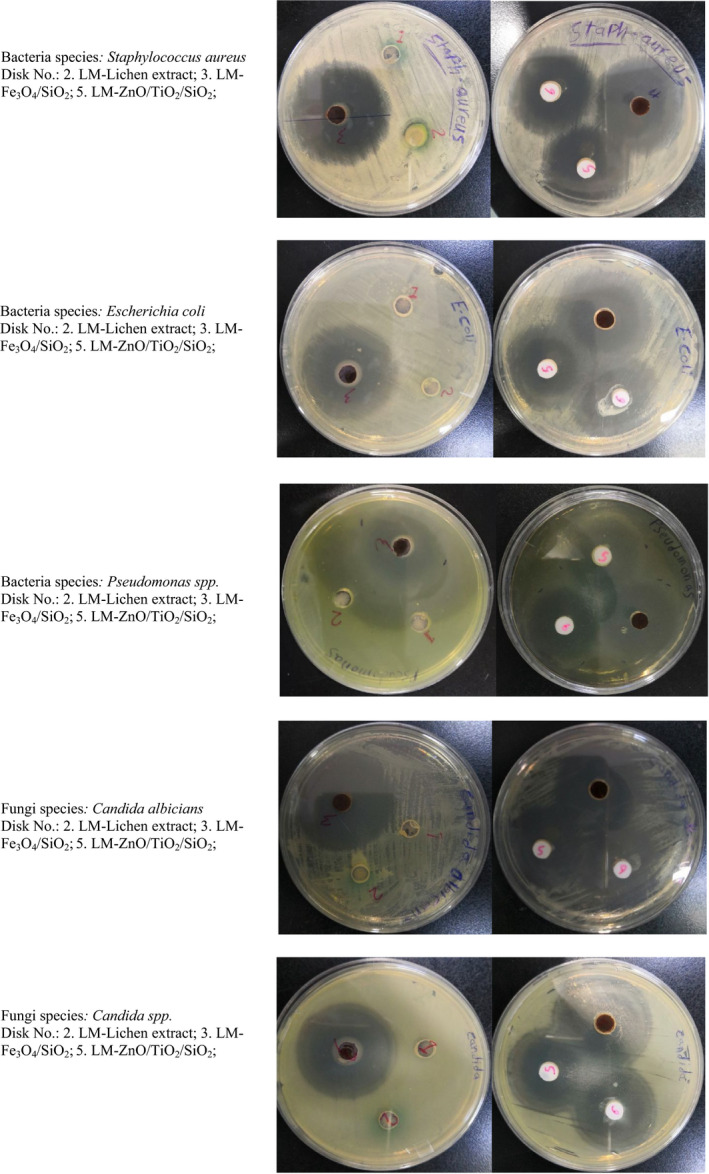
Antibacterial (Well diffusion method) and antifungal activities of LM lichen extract and green nanomaterials

Three pathogenic bacterial species (*Staphylococcus aureus*, *Escherichia coli*, and *Pseudomonas* spp.) and five pathogenic fungal species (*Candida albicians*, *Candida* spp., *Aspergillus flavus*,* Aspergillus niger*, and *Aspergillus terrus*) were used to examine antibacterial and antifungal activities of the prepared nanomaterials and aqueous extract of lichen species to compare antimicrobial activity between the natural aqueous extract of lichen and the laboratory‐prepared lichen‐derived nanomaterials, Tables 2 and 3. Also, the poisoned food method was used in the preliminary screening of lichen aqueous extracts and prepared nanomaterials for their antifungal properties’ evaluation, Figures 10 and 11. As it is visible in Figures 10 and 11 and also Tables 2 and 3, at the same conditions, the lichen extract demonstrated significantly less antibacterial and antifungal activities than green nanocomposites which probably refers to the large surface area of NCs and hyperaccumulation of bioactive phytochemicals on their surface. Although, the bioactivity of the Fe_3_O_4_/SiO_2_ NCs is more than ZnO/TiO_2_/SiO_2_ which possibly refers to the more deposition of bioactive phytochemicals of its surface than the ZnO/TiO_2_/SiO_2_ NCs. Beside the bioactivity, the adsorbed antioxidant phytochemicals on the surface of nanocomposites caused to their long‐term stabilization against decomposition and deformation reactions.

**Table 2:** The results obtained by disk diffusion method to evaluate the antibacterial activity

	Lichen Species	*Lecanora muralis*		
	Sample Number	2	3	5
	Inhibition zone (mm)	Lichen Extract	Fe_3_O_4_/SiO_2_	ZnO/TiO_2_/SiO_2_
Bacteria	*Staphylococcus aureus*	6 mm	39 mm	40 mm
	*Escherichia coli*	6 mm	35 mm	31 mm
	*Pseudomonas* spp.	6 mm	43 mm	37 mm
Fungi	*Candida albicians*	6 mm	35 mm	29 mm
	*Candida* spp.	6 mm	35 mm	31 mm

**Table 3:** The results obtained by well diffusion method to evaluate the antibacterial activity

	Lichen Species	*Lecanora muralis*		
	Sample Number	2	3	5
	Inhibition zone (mm)	Lichen Extract	Fe_3_O_4_/SiO_2_	ZnO/TiO_2_/SiO_2_
Bacteria	*Staphylococcus aureus*	6 mm	41 mm	40 mm
	*Escherichia coli*	6 mm	39 mm	39 mm
	*Pseudomonas*	6 mm	38 mm	37 mm
Fungi	*Candida albicians*	6 mm	44 mm	35 mm
	*Candida* spp.	6 mm	39 mm	31 mm

## 4 CONCLUSIONS

During this research, the ability of *Lecanora muralis (LM)* lichen aqueous extract was examined to biosynthesis of Fe_3_O_4_/SiO_2_ and ZnO/TiO_2_/SiO_2_ nanocomposites through a simple, eco‐friendly, economic, and rapid method as characterized using micrograph and diffractogram techniques. Analysis of the lichen extract using GC–Mass technique revealed the presence of valuable bioactive phytochemicals inside that. Also, the green synthesized nanocomposites showed a very good bioactivity against some common pathogenic bacteria and fungi due to the accumulation of lichen phytochemicals on the surface of them.

## CONFLICT OF INTEREST

The authors declare that they do not have any conflict of interest.

## ETHICAL APPROVAL

This study does not involve any human or animal testing.
